# Depth-specific responses to 8-week additional aquatic plyometric training on explosive performance and start-turn metrics in collegiate male swimmers

**DOI:** 10.3389/fphys.2026.1880612

**Published:** 2026-08-05

**Authors:** Mian Xu, Minyue Hou, Junsheng Cao, Minyu Hu

**Affiliations:** 1 Department of Physical Education, Nanjing Medical University, Nanjing, Jiangsu, China; 2 School of Physical Education, Shandong Normal University, Jinan, Shandong, China; 3 School of Physical Education, Jiangsu Second Normal University, Nanjing, Jiangsu, China

**Keywords:** aquatic plyometric training, countermovement jump, high-speed video, immersion depth, lower-limb explosive performance, mixed-effects model, swimming start, tumble turn

## Abstract

**Background:**

Aquatic plyometric training (APT) may improve explosive performance while reducing impact loading; however, the extent to which immersion depth influences transfer to swimming-specific start and turn performance remains unclear. This exploratory randomized study compared depth-specific responses to 8 weeks of additional aquatic plyometric-style training performed at different immersion depths in collegiate male swimmers who maintained regular swim training.

**Methods:**

Thirty collegiate male swimmers were randomized to shallow (knee/patella), mid-depth (waist/umbilicus), or deep (xiphoid/chest) APT groups. Participants completed two APT sessions per week for 8 weeks while maintaining regular swim training. Countermovement jump, squat jump, 15-m start time, turn 5-m in time, wall contact time, and turn 5-m out time were assessed before and after the intervention using high-speed video. Three trials were averaged for each outcome. Linear mixed-effects models were used to test group-by-time interactions, and within-rater reliability was assessed in a subsample of 10 participants.

**Results:**

Significant group-by-time interactions were observed for countermovement jump (p = 0.033), squat jump (p = 0.009), 15-m start time (p <0.001), and wall contact time (p = 0.016), indicating depth-dependent patterns of change. All groups showed favorable within-group changes in countermovement jump, squat jump, and 15-m start time, with the largest mean changes generally observed in the mid-depth group. Turn 5-m out time improved within groups, but the group-by-time interaction did not reach statistical significance (p = 0.095). No significant interaction was observed for turn 5-m in time (p = 0.457). Video-derived outcomes showed excellent within-rater reliability (ICC = 0.991-0.997; CV = 0.19%-1.40%).

**Conclusion:**

In collegiate male swimmers who maintained regular swim training, 8 weeks of additional APT was associated with favorable changes in lower-limb explosive performance and selected race-section metrics. Immersion depth appeared to influence the magnitude of adaptation, with the mid-depth condition showing larger mean changes in several outcomes, particularly compared with deep immersion, whereas differences between mid-depth and shallow immersion were less consistent. These findings should be interpreted as depth-specific responses to additional aquatic plyometric-style training performed alongside regular swim training, rather than as definitive evidence that APT alone caused the observed changes or is superior to other training approaches.

## Introduction

In competitive swimming, success is often determined by fractions of a second, with race starts and turns contributing significantly to overall performance (Marinho et al., 2020; Born et al., 2024). These critical race components are heavily dependent on the athlete’s ability to generate maximal explosive force with the lower limbs to propel the body from the starting block or the pool wall (West et al., 2011). Consequently, developing lower-body power is a primary objective of dry-land conditioning programs for swimmers (Hermosilla et al., 2021). Plyometric training (PT), which involves exercises that exploit the stretch-shortening cycle (SSC), is a well-established method for enhancing muscular power, jump performance, and sprint speed across various athletic populations (Markovic and Mikulic, 2010; Slimani et al., 2016).

However, traditional land-based plyometric training (LPT) is characterized by high-impact forces, which can lead to significant muscle soreness and an elevated risk of musculoskeletal injury, particularly in athletes already subjected to high training volumes, such as competitive swimmers (Robinson et al., 2004; Martel et al., 2005). This has prompted growing interest in aquatic plyometric training (APT) as a viable alternative. The buoyancy of water reduces effective body weight and, consequently, the ground reaction forces (GRF) experienced during jumping exercises, thereby mitigating exercise-induced muscle damage and soreness (Robinson et al., 2004; Arazi et al., 2016). Systematic reviews have concluded that APT is effective for improving strength, jumping, and sprinting performance, often yielding gains comparable to those of LPT (Mullenax et al., 2021; Heywood et al., 2022). Moreover, some studies have reported superior improvements in certain performance metrics following APT relative to LPT, particularly in volleyball players (Martel et al., 2005; Yu et al., 2025).

A critical, yet under-investigated, variable in APT prescription is water immersion depth. Immersion depth directly influences the biomechanical and physiological demands of aquatic exercise. Deeper immersion progressively reduces effective body weight and peak GRF while increasing total contact time during jumps (Dell’Antonio et al., 2016). However, if contact time becomes too prolonged, the plyometric effect may diminish, as the contribution of the stretch-shortening cycle (SSC) is reduced and the elastic energy stored may no longer be effectively utilized. Furthermore, greater fluid drag at deeper immersion levels may attenuate movement velocity yet simultaneously enhance concentric power development (Louder et al., 2016). This creates a potential trade-off: shallower depths offer a stimulus more specific to high-velocity, high-impact land-based movements, whereas deeper depths afford greater joint unloading and potentially greater resistance during the concentric phase. Mid-depth immersion (umbilicus/waist level) may therefore provide a practical balance between mechanical loading, aquatic resistance, and reduced landing impact. Previous research has compared different immersion depths (e.g., waist-deep versus chest-deep) and shown their influence on force, power, and jump kinematics (Miller et al., 2007; Donoghue et al., 2011), yet no study has directly examined the three standardized anatomical landmarks (patella, umbilicus, and xiphoid process) in swimmers. Nevertheless, it remains unclear which depth provides the most appropriate training stimulus for performance enhancement in swimmers, whose primary goal is to transfer gains in vertical explosive performance to horizontal propulsion in the water.

Previous research has compared APT with LPT (Stemm and Jacobson, 2007; Jurado-Lavanant et al., 2018) or examined APT performed on alternative surfaces such as sand (Sanchez-Ottado et al., 2025; Yu et al., 2025), and some investigations have evaluated APT at a single immersion depth in swimmers or other athletic cohorts (Dell'Antonio et al., 2022). Despite the growing adoption of APT in competitive swimming programs, coaches currently lack evidence-based guidance on the selection of optimal immersion depth, a parameter with direct implications for training load, injury risk, and the transfer of explosive power to swimming-specific performance. Resolving this question is essential for the rational prescription of APT and the optimization of dry-land conditioning in competitive swimmers.

Therefore, this exploratory study compared depth-specific responses to an 8-week additional aquatic plyometric-style training program performed at three immersion depths (shallow, mid-depth, and deep) on lower-limb explosive performance (countermovement jump and squat jump) and key freestyle start and turn metrics in collegiate male swimmers. We hypothesized that swimmers in all three APT conditions would show favorable pre-to-post changes, but that the magnitude of change would differ by immersion depth. Specifically, we expected the mid-depth condition to show larger mean changes in selected outcomes because it may provide a practical balance between mechanical loading, aquatic resistance, and reduced landing impact.

## Methods

### Study design

This study used an 8-week, three-arm, randomized parallel-group design to compare depth-specific responses to APT in collegiate male swimmers. Participants were assessed at baseline before the intervention (Pre, week 0) and immediately after the 8-week training period (Post, week 8). Post-intervention testing was conducted 3–5 days after the final training session to minimize the potential influence of acute fatigue. The study was designed primarily to compare different immersion-depth prescriptions within APT. No non-training control group or land-based plyometric training group was included; therefore, causal inferences regarding the isolated effect or superiority of APT over other training modes should be made cautiously.

All participants maintained their regular swimming training throughout the study period and completed two additional APT sessions per week. Intervention content, training frequency, sets, repetitions, rest intervals, and weekly progression were standardized across groups. The only planned experimental factor that differed among groups was water immersion depth during APT.

### Participants

Thirty male collegiate swimmers were recruited from a university swimming team. All participants were regular members of the university swimming team and were trained competitive swimmers. To further characterize the competitive level of the sample, all participants had achieved at least the Chinese National First-Class Athlete standard in the 100-m freestyle, with personal-best 100-m freestyle times of 55.50 s or faster before enrollment. This criterion was used to ensure that the sample represented trained competitive swimmers rather than recreationally trained individuals. Participants were eligible if they were 18–25 years old, had at least 2 years of systematic swimming training experience, had no lower-limb injury during the preceding 3 months, and were able to perform maximal competitive starts and freestyle flip turns. Before baseline data collection, all participants completed a familiarization session. During this session, participants were introduced to the testing procedures, camera positioning, event definitions, and the execution requirements for the countermovement jump, squat jump, 15-m start, and freestyle turn tests. Participants practiced each testing task at submaximal intensity and then performed practice trials until they could complete the procedures with stable technique. They were also familiarized with the aquatic plyometric drills and the required arm-position restrictions before the intervention began. No data from the familiarization session were used in the formal analysis. Regarding previous training background, all participants had experience with regular dry-land conditioning as part of their university team training. This included general strength and conditioning exercises, but none of the participants had participated in a structured lower-limb plyometric training program more than once per week during the 4 weeks before enrollment. During the intervention period, participants were instructed not to add any new strength or plyometric training outside the prescribed team training and APT program. Regular swimming training and dry-land conditioning exposure were monitored by the coaching staff to minimize between-group differences in concomitant training.

All participants were informed of the study procedures, potential risks, and benefits before participation, and written informed consent was obtained. The study was approved by the Shandong Normal University Institutional Review Board (approval number: SDNUTYDW2024089), and all procedures adhered to the Declaration of Helsinki.

### Sample size considerations

Because the intervention was conducted within a single university swimming team, the available sample was constrained by team roster size and training feasibility. No formal *a priori* sample-size calculation was performed. Accordingly, this study should be interpreted as an exploratory randomized training study, and the precision of between-group estimates should be considered when interpreting nonsignificant or borderline findings.

### Randomization and allocation

After baseline testing, participants were stratified by baseline 15-m start performance and then randomly assigned in a 1:1:1 ratio to one of three APT groups: shallow immersion (LOW), mid-depth immersion (MID), or deep immersion (HIGH). Randomization was performed with a computer-generated random sequence by an investigator who was not involved in participant testing or training supervision. Allocation was concealed until baseline assessments had been completed.

### Training intervention

The APT intervention lasted 8 weeks and was performed twice per week, with at least 48 h between sessions (**Table 1A**). Each APT session was performed after the swimmers’ regular swimming training session on the same training day, rather than before or during the main swimming session. This arrangement was used to avoid acute fatigue from APT influencing the quality of the regular swimming training. Each session lasted approximately 25–35 min and included a standardized warm-up, main plyometric training set, and cool-down.

**Table 1A:** Weekly dose targets and session scheduling.

Week	Sessions/week	Target foot contacts/session	Recommended schedule	Main emphasis
1–2	2	36–42	A + B	Technique, landing control, low amplitude
3–4	2	42–48	A + B	Volume build, introduce lateral/horizontal work
5–6	2	48–54	A + B	Higher neuromuscular demand, add unilateral (low amplitude)
7–8	2	50–56	A + B	Sport-oriented intent, rapid eccentric-to-concentric transitions where feasible

Each session began with an 8–10 min aquatic warm-up consisting of light aquatic jogging and dynamic mobility exercises. The main training section lasted approximately 15–20 min and included standardized jump-based aquatic exercises, including pogo jumps, explosive countermovement-style jumps, lateral or horizontal jumping tasks, and low-amplitude unilateral hops. Each session ended with a 3–5 min cool-down consisting of easy aquatic walking and stretching. In the present study, the term aquatic plyometric training was used operationally to describe jump-based exercises performed with maximal explosive intent in water, rather than to imply that these exercises were mechanically identical to land-based plyometric training. Because buoyancy alters the eccentric, landing, and contact characteristics of jumping, this distinction is particularly relevant at deeper immersion levels, where the reduction in effective body weight may attenuate the rapid eccentric loading normally associated with a classic stretch-shortening cycle. Therefore, participants were instructed to perform each countermovement-style jump with the fastest possible eccentric-to-concentric transition while maintaining technical quality, but the actual mechanical stimulus was expected to vary across immersion-depth conditions (**Table 1B**).

**Table 1B:** Standardized session structure and drill categories across the 8-week intervention.

Component	Week 1–2	Week 3–4	Week 5–6	Week 7–8	Notes
Aquatic warm-up	8–10 min	8–10 min	8–10 min	8–10 min	Aquatic jogging and dynamic mobility
Vertical ankle stiffness drills	Pogo jumps	Pogo jumps	Pogo jumps	Pogo jumps	Emphasize short contact and alignment
Bilateral explosive jumps	CMJ-style jumps	CMJ-style jumps	CMJ-style jumps	CMJ-style jumps	Maximal intent with controlled landing
Lateral/horizontal jumps	Introduced at low amplitude	Moderate volume	Moderate-to-high intent	Sport-oriented intent	Emphasize lateral and horizontal force application
Unilateral low-amplitude hops	Technique-focused	Low volume	Progressed volume	Maintained volume	Regressed if technique deteriorated
Cool-down	3–5 min	3–5 min	3–5 min	3–5 min	Easy walking and stretching
Total foot contacts	36–42	42–48	48–54	50–56	Total contacts were constrained within the weekly target range

Training volume was standardized according to total foot contacts. The weekly training dose was progressively increased across the 8-week period. During weeks 1-2, participants completed 36–42 contacts per session, emphasizing technical adaptation and landing control. During weeks 3-4, volume increased to 42–48 contacts per session. During weeks 5-6, participants completed 48–54 contacts per session, with increased neuromuscular demand. During weeks 7-8, volume was maintained at 50–56 contacts per session, with greater emphasis on sport-oriented explosive intent and rapid eccentric-to-concentric transitions where mechanically feasible within each immersion-depth condition.

Participants were instructed to perform each jump with maximal intent while maintaining proper technique, including neutral trunk alignment, controlled landing, and appropriate lower-limb alignment. If technical quality deteriorated, such as excessive knee valgus, poor landing control, or inability to maintain movement quality, the exercise was regressed by reducing movement amplitude, replacing unilateral drills with bilateral alternatives, or reducing repetitions while keeping the planned training volume as close as possible.

To minimize the potential confounding effect of arm assistance during aquatic jumping, arm action was standardized during all APT drills. Participants were instructed to keep their hands on their hips or crossed in front of the trunk throughout each jump. Arm swing, sculling motions, or other deliberate arm movements used to generate additional lift or balance were not permitted. All sessions were supervised by qualified staff, and arm position was monitored continuously during the drills. If obvious arm assistance, sculling, or loss of the prescribed arm position was observed, participants were immediately corrected, and the repetition was repeated or the exercise was regressed to a lower-amplitude bilateral variation. This procedure was used to maintain a lower-limb-dominant plyometric stimulus across immersion-depth conditions.

### Immersion depth conditions

Immersion depth was standardized using anatomical landmarks to account for individual differences in body height. Participants in the shallow immersion group trained with the water level approximately at the patella or knee. Participants in the mid-depth immersion group trained with the water level approximately at the umbilicus or waist. Participants in the deep immersion group trained with the water level approximately at the xiphoid process or chest.

The training was conducted in designated areas of the swimming pool corresponding to the assigned immersion-depth condition. Before each session, water depth was checked by the research staff, and each participant’s assigned immersion level was confirmed according to the relevant anatomical landmark while standing upright in the training area. Participants remained in their assigned pool area throughout the aquatic plyometric training session to maintain a consistent immersion depth. If the water level did not correspond to the assigned anatomical landmark, the participant’s position within the pool area was adjusted before training began.

**Figure 1:**
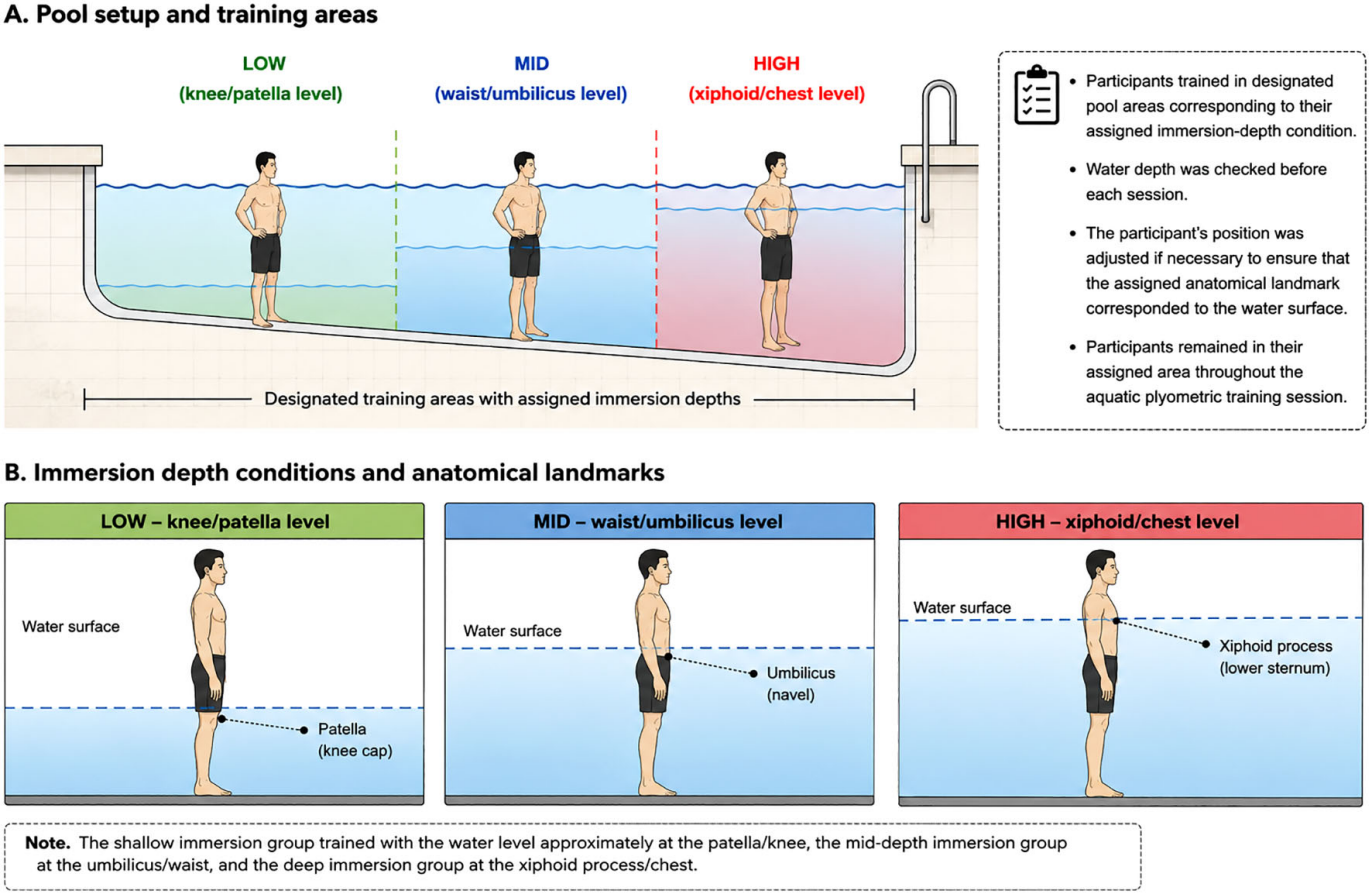
Schematic illustration of the aquatic plyometric training setup and immersion-depth standardization. Panel **(A)** shows the pool setup and designated training areas for the three immersion-depth conditions. Participants trained in pool areas corresponding to their assigned condition: LOW, shallow immersion at approximately the knee/patella level; MID, mid-depth immersion at approximately the waist/umbilicus level; and HIGH, deep immersion at approximately the xiphoid/chest level. Water depth was checked before each training session, and the participant’s position was adjusted when necessary to ensure that the assigned anatomical landmark corresponded to the water surface. Participants remained in their assigned area throughout the aquatic plyometric training session. Panel **(B)** illustrates the anatomical landmarks used to standardize immersion depth across participants. The image is schematic and not drawn to scale.

A schematic illustration of the experimental setup, including the three immersion-depth conditions and the anatomical landmarks used for standardization, is provided in **Figure 1**.

### Control of concomitant training and safety monitoring

All swimmers continued their regular swimming training under the supervision of team coaches in a standard 50-m swimming pool. Weekly swimming volume and the number of high-intensity swimming sessions were recorded throughout the intervention to monitor potential between-group differences in concomitant training exposure. These variables were compared across groups and considered when interpreting the intervention effects.

Training attendance was recorded for every APT session. Participants were considered compliant with the intervention if they completed at least 85% of the prescribed APT sessions, corresponding to at least 14 of the 16 total sessions. Participants who failed to meet this attendance criterion or who missed post-intervention testing would have been excluded from the per-protocol analysis. Session rating of perceived exertion was recorded after each session using a 0–10 scale. Delayed-onset muscle soreness was also recorded using a 0–10 scale.

### Outcome measures and testing procedures

All outcome measures were assessed before and after the 8-week intervention. For each test, participants completed three maximal trials, and the mean of the three trials was used for statistical analysis. The 15-m start time was considered the primary swimming-specific outcome because it is commonly used in swimming start research and represents a race-relevant indicator of start performance (West et al., 2011). CMJ and SJ were selected as secondary outcomes because they are widely used field tests for assessing lower-limb explosive performance and neuromuscular function. Wall contact time, turn 5-m in time, and turn 5-m out time were also treated as secondary outcomes because segment-based turn analyses are commonly used to characterize the approach, wall-contact, and exit phases of freestyle tumble turns (David et al., 2022; Puel et al., 2023).

### Testing schedule and standardization

All performance tests were conducted in the same indoor swimming facility under standardized conditions before and after the 8-week intervention. To minimize the potential influence of fatigue, testing was scheduled after adequate recovery from previous training sessions, and participants were instructed to avoid additional strenuous exercise before testing. Post-intervention testing was conducted 3–5 days after the final APT session. For each participant, pre- and post-intervention tests were performed at a similar time of day and using the same testing order, equipment setup, camera position, and verbal instructions.

Before formal testing, participants completed a standardized warm-up consisting of 5–8 min of light jogging or easy swimming, dynamic mobility exercises, and several progressive practice trials specific to the subsequent test. The testing order was fixed for all participants: lower-limb explosive performance tests were performed first, followed by the 15-m start test and then the freestyle turn test. Adequate recovery was provided between trials and between test sections to reduce the influence of acute fatigue on performance.

### Lower-limb explosive performance

Lower-limb explosive performance was assessed using the countermovement jump (CMJ) and squat jump (SJ). These tests are commonly used field measures of lower-limb explosive performance and neuromuscular function. Jump performance was quantified from flight time derived from high-speed video.

For dry-land jump analyses, a high-speed action camera was positioned laterally at approximately hip height and 3–5 m from the participant to ensure full-body capture. Video was recorded at 120 frames per second, corresponding to a temporal resolution of approximately 8.33 ms per frame, and was analyzed frame by frame using Kinovea software. Jump height was calculated from flight time using the following equation: h = (g × t²)/8, where h is jump height, g is gravitational acceleration (9.81 m·s^-^²), and t is flight time. Flight time was determined from take-off, defined as the first frame in which both feet were off the ground, to landing, defined as the first frame in which either foot contacted the ground.

Both tests were performed with the hands placed on the hips to minimize the contribution of arm swing. For the SJ, participants held a static squat position with approximately 90° of knee flexion for approximately 2 s before take-off to minimize countermovement. The test administrator visually monitored the starting position and movement execution, and the high-speed video was checked frame by frame to verify that no obvious countermovement occurred before take-off. Trials were considered invalid and repeated if participants showed additional downward displacement, further hip or knee flexion, a preparatory bouncing action, or any other visible countermovement before take-off. For the CMJ, participants performed a rapid downward movement followed immediately by a maximal vertical jump. Trials were separated by 60–120 s of rest.

### Swimming start performance

Swimming start performance was assessed using a maximal competitive start to 15 m. Each participant completed three maximal starts, with 3–5 min of rest between trials. For start analysis, a high-speed action camera was positioned poolside, above the water surface, and perpendicular to the swimming lane to minimize parallax error. The camera position and distance were kept consistent across pre- and post-intervention testing. Clearly visible 15-m markers were used to define the timing plane (West et al., 2011).

The primary start outcome was 15-m start time from block take-off. This outcome was derived solely from frame-by-frame video analysis and was calculated from the first frame in which both feet left the starting block to the first frame in which the swimmer’s head crossed the 15-m marker. No manual stopwatch timing was used as either the primary or backup timing method.

Reaction time was not included in the 15-m start outcome because the purpose of this study was to evaluate the movement and propulsion-related components of start performance after block take-off, which were considered more directly relevant to the lower-limb training intervention. Therefore, the reported 15-m start time should be interpreted as the time from block take-off to 15 m rather than as the total time from the acoustic start signal to 15 m. When the 5-m marker was clearly visible, 0–5 m and 5–15 m split times were also calculated to support interpretation of start performance changes.

### Freestyle turn performance

Turn performance was assessed using a standardized freestyle flip turn. Clearly visible 5-m markers before and after the wall were used to define turn-related timing segments, consistent with segment-based analyses of swimming turn performance (Puel et al., 2023). Each participant performed three maximal turn trials with adequate recovery between trials.

For turn analysis, a high-speed action camera was positioned poolside, above the water surface, and perpendicular to the swimming lane. The same camera position was used during pre- and post-intervention testing. All turn-related timing outcomes were derived solely from frame-by-frame video analysis, and no manual stopwatch timing was used.

The following turn outcomes were analyzed: 5-m in time, wall contact time, and 5-m out time. The 5-m in time was calculated from the first frame in which the swimmer’s head crossed the 5-m-in marker to the first frame of foot contact with the wall. Wall contact time was calculated from the first frame of foot contact with the wall to the first frame of foot-off. The 5-m out time was calculated from the first frame of foot-off to the first frame in which the swimmer’s head crossed the 5-m-out marker.

### High-speed video capture and analysis

All video files were analyzed frame by frame by the same assessor using Kinovea software. To evaluate within-rater reliability of the video-derived outcomes, a random subsample of 10 participants was reanalyzed after a 7-day interval. Intraclass correlation coefficients were calculated using a two-way mixed-effects absolute-agreement model. Single-measure reliability and reliability based on the mean of repeated trials were reported. Absolute reliability was quantified using the coefficient of variation and typical error.

### Statistical analysis

Data were inspected for normality using the Shapiro-Wilk test and for homogeneity of variance before inferential analysis. Descriptive statistics are presented as mean ± standard deviation. Baseline between-group differences were examined using one-way analysis of variance to evaluate the comparability of the randomized groups.

The primary analysis used linear mixed-effects models. Group (LOW, MID, and HIGH), time (Pre and Post), and the group-by-time interaction were included as fixed effects, and participant was included as a random intercept to account for repeated measures. The group-by-time interaction was used to determine whether pre-to-post changes differed among immersion-depth conditions. Given the small sample and the absence of statistically significant baseline differences among groups, unadjusted models were used as the primary analysis.

Within-group changes from pre- to post-intervention were summarized descriptively using mean changes, 95% confidence intervals, and paired comparisons. Between-group differences in change scores were explored when significant or meaningful group-by-time interactions were observed. Standardized effect sizes were added to aid interpretation. For within-group pre-to-post changes, Cohen’s dz was calculated as the mean change divided by the standard deviation of the change scores. For the Group × Time interaction, partial eta squared (ηp²) was calculated from equivalent two-way mixed ANOVA models with group as the between-subject factor and time as the within-subject factor. These interaction effect sizes were reported to complement the mixed-model p values and to facilitate interpretation of practical magnitude. Because several secondary outcomes and pairwise comparisons were examined, these analyses were considered exploratory. The Group × Time interaction from the linear mixed-effects model was retained as the primary test for depth-specific differences. Exploratory pairwise comparisons of change scores were performed only when the Group × Time interaction was significant or considered meaningful and were adjusted using the Holm-Bonferroni method. Within-group pre-to-post comparisons were descriptive and exploratory. Statistical significance was set at p < 0.05.

## Results

**Figure 2:**
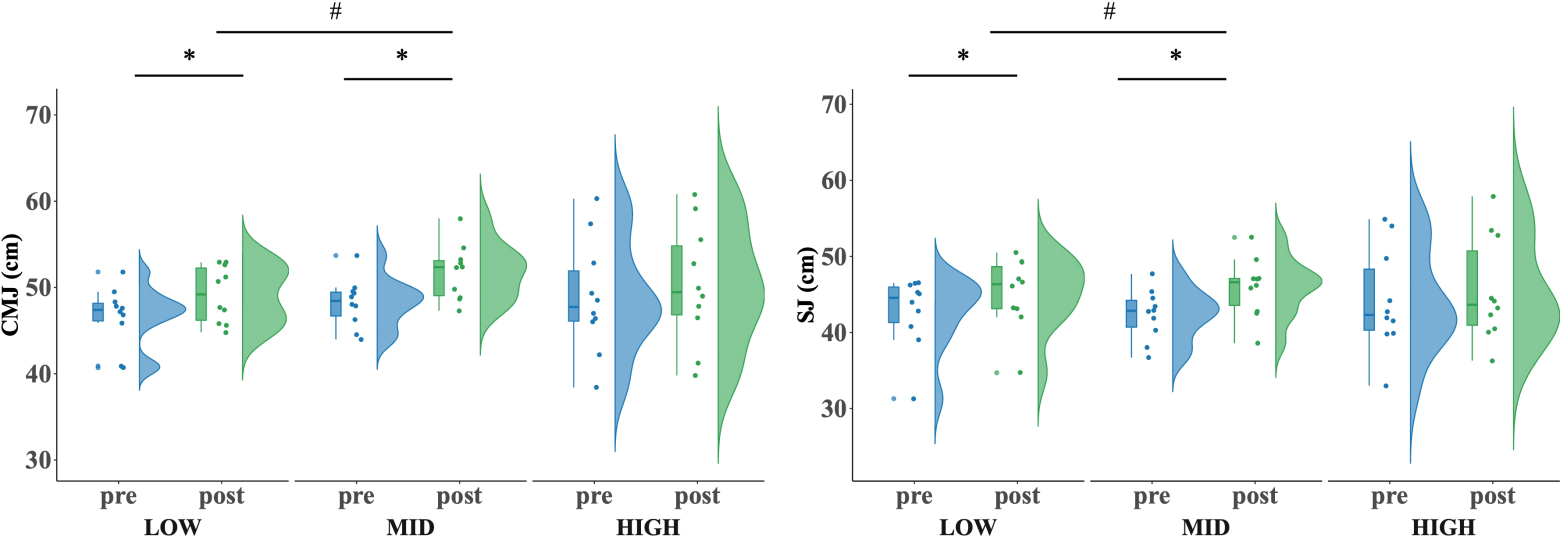
Pre-to-post changes in lower-limb explosive performance across immersion-depth conditions. Raincloud plots show individual participant data, boxplots, and distribution densities for countermovement jump (CMJ) and squat jump (SJ) before and after the 8-week intervention. Each dot represents one participant’s mean value calculated from three trials. LOW indicates shallow immersion at the knee/patella level; MID, mid-depth immersion at the waist/umbilicus level; and HIGH, deep immersion at the xiphoid/chest level. Higher values indicate better jump performance. * indicates a significant within-group pre-to-post change (p < 0.05). # indicates a significant between-group difference in change scores in exploratory Holm-Bonferroni-adjusted comparisons (p < 0.05).

**Figure 3:**
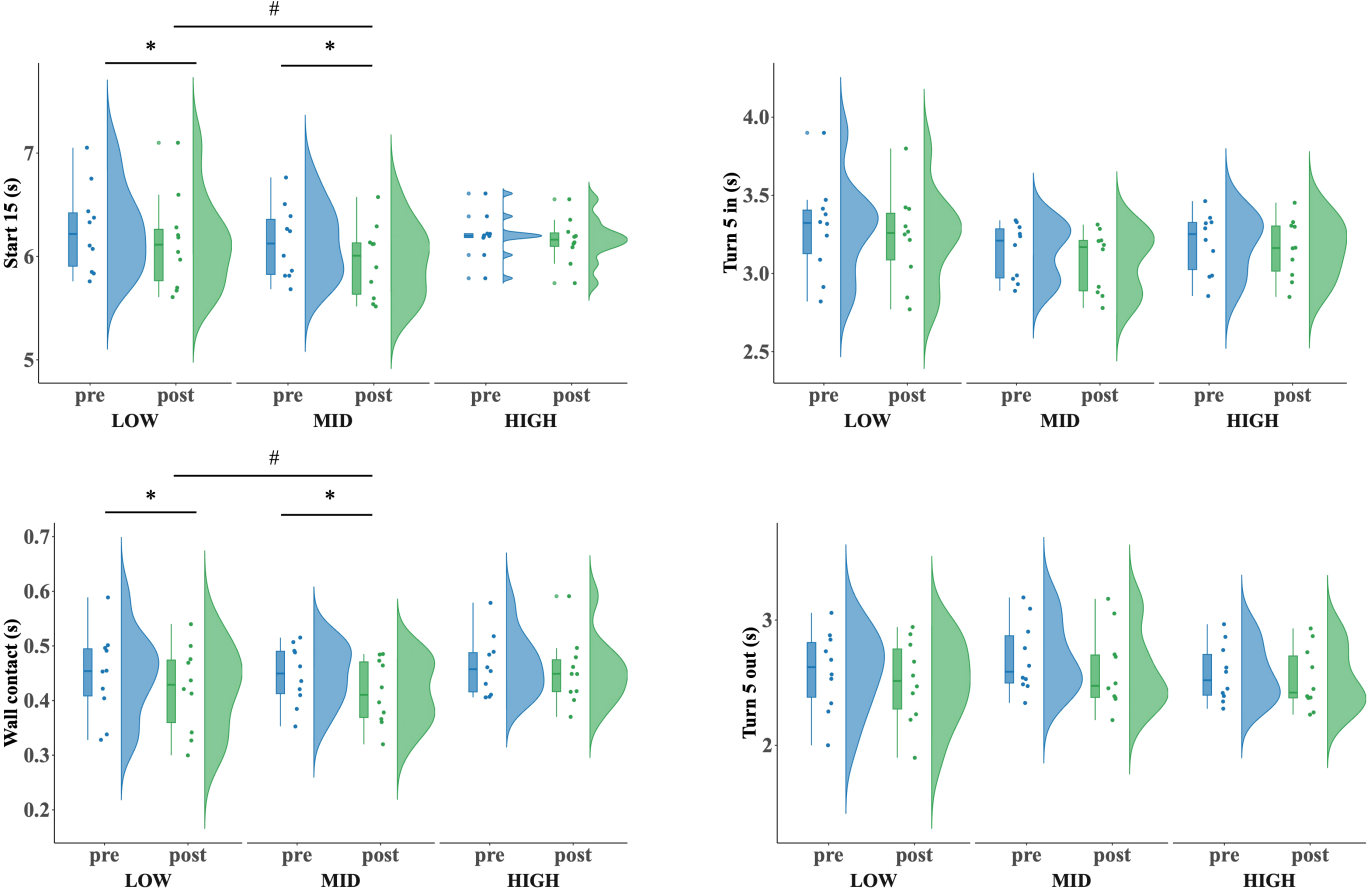
Pre-to-post changes in swimming start and turn performance across immersion-depth conditions. Raincloud plots show individual participant data, boxplots, and distribution densities for 15-m start time, turn 5-m in time, wall contact time, and turn 5-m out time before and after the 8-week intervention. Each dot represents one participant’s mean value calculated from three trials. LOW indicates shallow immersion at the knee/patella level; MID, mid-depth immersion at the waist/umbilicus level; and HIGH, deep immersion at the xiphoid/chest level. Start 15 refers to the time from block take-off to the swimmer’s head crossing the 15-m marker. Turn 5 m in refers to the time from the swimmer’s head crossing the 5-m-in marker to first foot contact with the wall. Wall contact refers to the time from first foot contact with the wall to foot-off. Turn 5 m out refers to the time from foot-off to the swimmer’s head crossing the 5-m-out marker. Lower values indicate better performance for all time-based outcomes. * indicates a significant within-group pre-to-post change (p < 0.05). # indicates a significant between-group difference in change scores in exploratory Holm-Bonferroni-adjusted comparisons (p < 0.05).

All participants completed the study, and all randomized swimmers were included in the final analysis. All participants met the predefined attendance criterion of completing at least 14 of the 16 prescribed APT sessions, and no participant was excluded because of insufficient training compliance. Training adherence was high across the three groups (attendance out of 16 sessions: LOW, 15.2 ± 0.6; MID, 15.5 ± 0.7; HIGH, 14.8 ± 0.8 sessions). Baseline characteristics and training exposure are summarized in **Table 2**. Average weekly swim volume and the number of high-intensity swim sessions were comparable among groups (all p > 0.05), although high-intensity session frequency showed a nonsignificant trend (p = 0.096). Session RPE and delayed-onset muscle soreness were low to moderate across the intervention and did not differ significantly among groups. No slips, musculoskeletal injuries, or adverse events requiring medical attention were reported during the 8-week APT program. Because all participants continued regular swim training and no external control condition was included, the following results are presented as pre-to-post changes observed during regular swim training plus additional APT. Group-by-time interactions were used to examine whether the magnitude of change differed among immersion-depth conditions, rather than to infer the isolated effect of APT (**Table 3**). Pre-to-post changes in lower-limb explosive performance and swimming start and turn performance are presented in **Figures 2** and **3**, respectively.

**Table 2:** Baseline characteristics and training exposure (mean ± SD).

Variable	Low	Mid	High	p (ANOVA)
Age (y)	19.76 ± 1.17	19.99 ± 0.94	20.15 ± 1.21	0.737
Height (m)	1.78 ± 0.06	1.79 ± 0.06	1.82 ± 0.05	0.287
Body mass (kg)	74.51 ± 8.66	74.98 ± 4.88	72.75 ± 3.49	0.692
Attendance (0–16)	15.20 ± 0.63	15.50 ± 0.71	14.80 ± 0.79	0.107
Weekly swim volume (km)	26.63 ± 3.80	26.85 ± 3.73	28.99 ± 4.11	0.339
High-intensity sessions (/wk)	2.82 ± 0.85	2.99 ± 0.58	3.41 ± 0.56	0.096
CMJ (cm)	46.65 ± 3.48	48.21 ± 2.83	48.83 ± 6.59	0.532
SJ (cm)	42.75 ± 4.74	42.36 ± 3.32	42.19 ± 4.87	0.953
15-m start (s)	6.26 ± 0.42	6.13 ± 0.35	6.20 ± 0.21	0.717
Turn 5-m in (s)	3.22 ± 0.31	3.15 ± 0.20	3.21 ± 0.15	0.792
Wall contact (s)	0.45 ± 0.08	0.45 ± 0.06	0.46 ± 0.06	0.847
Turn 5-m out (s)	2.69 ± 0.30	2.71 ± 0.19	2.68 ± 0.25	0.958

Values are mean ± SD. Between-group differences were examined using one-way ANOVA. CMJ, countermovement jump; SJ, squat jump.

**Table 3:** Effects of 8-week aquatic plyometric training at different immersion depths.

Outcome	Grp	Pre (mean ± SD)	Post (mean ± SD)	Δ Post–Pre (95% CI)	Within p	Cohen’s dz	G×T p	ηp² (G×T)
CMJ (cm)	LOW	46.65 ± 3.48	49.16 ± 3.24	2.51 (0.97, 4.05)	0.005	1.164	0.033	0.203
CMJ (cm)	MID	48.21 ± 2.83	51.79 ± 3.21	3.58 (2.24, 4.92)	<0.001	1.917	0.033	0.203
CMJ (cm)	HIGH	48.83 ± 6.59	50.24 ± 6.96	1.41 (0.37, 2.45)	0.014	0.967	0.033	0.203
SJ (cm)	LOW	42.75 ± 4.74	45.17 ± 4.66	2.42 (1.15, 3.69)	0.002	1.359	0.009	0.270
SJ (cm)	MID	42.36 ± 3.32	45.92 ± 3.89	3.56 (2.67, 4.45)	<0.001	2.870	0.009	0.270
SJ (cm)	HIGH	44.16 ± 6.82	45.50 ± 6.89	1.34 (0.17, 2.51)	0.029	0.818	0.009	0.270
15-m start (s)	LOW	6.257 ± 0.419	6.136 ± 0.459	-0.121 (-0.188, -0.055)	0.002	-1.311	0.001	0.356
15-m start (s)	MID	6.135 ± 0.354	5.954 ± 0.353	-0.181 (-0.247, -0.115)	<0.001	-1.964	0.001	0.356
15-m start (s)	HIGH	6.201 ± 0.213	6.155 ± 0.220	-0.046 (-0.073, -0.019)	0.004	-1.206	0.001	0.356
Turn 5-m in (s)	LOW	3.288 ± 0.305	3.233 ± 0.297	-0.055 (-0.076, -0.033)	<0.001	-1.799	0.457	0.051
Turn 5-m in (s)	MID	3.141 ± 0.178	3.079 ± 0.198	-0.063 (-0.104, -0.022)	0.007	-1.100	0.457	0.051
Turn 5-m in (s)	HIGH	3.194 ± 0.197	3.159 ± 0.192	-0.035 (-0.082, 0.012)	0.124	-0.536	0.457	0.051
Wall contact (s)	LOW	0.448 ± 0.079	0.422 ± 0.079	-0.025 (-0.039, -0.011)	0.003	-1.267	0.016	0.241
Wall contact (s)	MID	0.447 ± 0.055	0.415 ± 0.059	-0.032 (-0.041, -0.022)	<0.001	-2.444	0.016	0.241
Wall contact (s)	HIGH	0.464 ± 0.056	0.453 ± 0.061	-0.011 (-0.022, -0.000)	0.044	-0.740	0.016	0.241
Turn 5-m out (s)	LOW	2.592 ± 0.320	2.510 ± 0.332	-0.082 (-0.138, -0.025)	0.010	-1.036	0.095	0.145
Turn 5-m out (s)	MID	2.696 ± 0.283	2.595 ± 0.315	-0.101 (-0.142, -0.060)	<0.001	-1.775	0.095	0.145
Turn 5-m out (s)	HIGH	2.571 ± 0.231	2.528 ± 0.249	-0.043 (-0.075, -0.010)	0.015	-0.942	0.095	0.145

Values are mean ± SD. Δ = Post − Pre. Within-group p values are descriptive paired comparisons. Group × Time p values are from linear mixed-effects models with random intercepts for participants. Pairwise change-score comparisons, when reported in the Results text, were exploratory and adjusted using the Holm-Bonferroni method. CI, confidence interval; CMJ, countermovement jump; SJ, squat jump.

For the primary swimming-specific outcome, 15-m start time was observed to decrease from Pre to Post in all three groups. Specifically, 15-m start time decreased from 6.257 ± 0.419 s to 6.136 ± 0.459 s in LOW (Δ = -0.121 s, 95% CI -0.188 to -0.055; p = 0.003), from 6.135 ± 0.354 s to 5.954 ± 0.353 s in MID (Δ = -0.181 s, 95% CI -0.247 to -0.115; p < 0.001), and from 6.201 ± 0.213 s to 6.155 ± 0.220 s in HIGH (Δ = -0.046 s, 95% CI -0.073 to -0.019; p = 0.004). In exploratory Holm-Bonferroni-adjusted change-score comparisons, the mean reduction in MID was larger than that in HIGH (Δ = -0.135 s, 95% CI -0.204 to -0.067; p = 0.001), whereas the mean reduction in HIGH was smaller than that in LOW (Δ = 0.076 s, 95% CI 0.007 to 0.145; p = 0.034). These follow-up comparisons suggest a larger mean change in the MID condition, particularly compared with HIGH, but should be interpreted as exploratory.

For turn performance, wall contact time showed a significant Group × Time interaction. Wall contact time decreased from 0.448 ± 0.079 s to 0.423 ± 0.079 s in LOW (Δ = -0.025 s; p = 0.003), from 0.447 ± 0.055 s to 0.415 ± 0.059 s in MID (Δ = -0.032 s; p < 0.001), and from 0.464 ± 0.056 s to 0.453 ± 0.061 s in HIGH (Δ = -0.011 s; p = 0.044). Exploratory Holm-Bonferroni-adjusted change-score comparisons indicated that the mean reduction in wall contact time was larger in MID than in HIGH (Δ = -0.021 s, 95% CI -0.034 to -0.008; p = 0.004). For 5-m out time, within-group reductions were observed in LOW (Δ = -0.082 s; p = 0.010), MID (Δ = -0.101 s; p < 0.001), and HIGH (Δ = -0.043 s; p = 0.015). However, the Group × Time interaction did not reach statistical significance (p = 0.095), indicating that the magnitude of change did not differ significantly among immersion-depth conditions. Therefore, no depth-specific conclusion was drawn for 5-m out time. Similarly, although 5-m in time decreased within all three groups, the Group × Time interaction was not significant (p = 0.457), and no depth-specific difference was inferred for this outcome (**Table 2** and **Figure 3**).

For lower-limb explosive performance, significant Group × Time interactions were observed for both CMJ and SJ. CMJ increased from 46.6 ± 3.5 cm to 49.2 ± 3.2 cm in LOW (Δ = 2.51 cm, 95% CI 0.97 to 4.05; p = 0.005), from 48.2 ± 2.8 cm to 51.8 ± 3.2 cm in MID (Δ = 3.58 cm, 95% CI 2.24 to 4.92; p < 0.001), and from 48.8 ± 6.6 cm to 50.2 ± 7.0 cm in HIGH (Δ = 1.41 cm, 95% CI 0.37 to 2.45; p = 0.014). Exploratory Holm-Bonferroni-adjusted change-score comparisons indicated that the mean increase in CMJ was larger in MID than in HIGH (Δ = 2.17 cm, 95% CI 0.59 to 3.75; p = 0.010). Similarly, SJ increased in LOW (Δ = 2.42 cm; p = 0.002), MID (Δ = 3.56 cm; p < 0.001), and HIGH (Δ = 1.34 cm; p = 0.029), with a larger mean increase in MID than in HIGH in exploratory change-score comparisons (Δ = 2.22 cm, 95% CI 0.85 to 3.59; p = 0.003). Overall, the MID condition showed larger mean changes in CMJ, SJ, 15-m start time, and wall contact time, particularly compared with the HIGH condition, whereas differences between MID and LOW were less consistent (**Table 2** and **Figure 2**).

Within-rater reliability for the video-derived outcomes, assessed in a subsample of 10 participants, was excellent across all key variables. ICC(A,1) values ranged from 0.991 to 0.997, and ICC(A,2) values ranged from 0.996 to 0.999. Absolute reliability was also high, as indicated by low coefficients of variation (CV range, 0.19%–1.40%) and small typical errors (e.g., Start15_s TE = 0.012 s; Wall Contact_s TE = 0.006 s), supporting the consistency of the video-based analysis.

## Discussion

This study compared depth-specific responses to 8 weeks of additional APT performed at different immersion depths on lower-limb explosive performance and freestyle start and turn performance in male collegiate swimmers who continued their regular swim training. The main finding was that favorable changes were observed across all three depth conditions, but the magnitude of adaptation differed by immersion depth. The waist-deep condition showed larger mean changes in several outcomes, particularly compared with the deep-immersion condition, including larger mean improvements in CMJ and SJ performance, greater reductions in 15-m start time, and more pronounced decreases in wall contact time. These findings suggest that immersion depth is a meaningful programming variable that may influence how APT-related adaptations are reflected in swimming-relevant performance outcomes. However, because this study did not include a non-training or land-based comparison group, the results should be interpreted primarily as evidence of depth-specific differences within APT rather than as evidence that APT alone caused all observed improvements.

The observed changes in start- and turn-related outcomes suggest that additional APT may be a useful supplementary strategy within a broader swim-training program. This finding is consistent with previous studies showing that plyometric training can improve lower-limb power and neuromuscular function, which are relevant to swimming start and turn ability (Pupišová et al., 2019; Kons et al., 2023). Prior research has also reported that aquatic plyometric exercise may enhance explosive performance while imposing less mechanical stress than traditional land-based plyometric training (Mullenax et al., 2021). The effectiveness of APT may be related to the unique loading characteristics of water. Buoyancy reduces effective body weight during landing and may attenuate impact forces, which could help reduce musculoskeletal stress and landing-related soreness or injury risk (Mullenax et al., 2021). At the same time, depending on immersion depth and the proportion of body segments moving through the water, aquatic resistance during the upward phase of jumping may contribute to concentric loading and support explosive force production. This combination of reduced eccentric impact and maintained concentric demand may be advantageous for swimmers, who require power development with minimal orthopedic burden. Nevertheless, because all groups continued regular swimming training, the present within-group improvements should be interpreted as the combined result of regular training plus additional APT rather than the isolated effect of APT alone.

Different immersion depths appeared to differentially influence lower-limb explosive adaptations. Although all three groups showed favorable pre-to-post changes, the waist-deep condition produced the largest mean gains in countermovement jump and squat jump performance and was superior to the deep-water condition in exploratory change-score comparisons. A likely explanation is that immersion depth alters the external loading characteristics of jumping exercise in water. Previous force-platform and biomechanical studies indicate that water depth can modify force requirements, contact characteristics, and landing impact during explosive movements (Louder et al., 2016; Pascoe and Cheslett, 2024). Greater immersion can reduce landing impact and effective body weight, but excessive unloading may also reduce the stretch-shortening cycle stimulus required for maximal plyometric adaptation (Waldvogel et al., 2021). In contrast, shallow immersion may preserve effective loading and faster stretch-shortening cycle characteristics, but the contribution of aquatic drag during the concentric phase may be more limited because a smaller proportion of the moving body mass is immersed. Waist-deep immersion may therefore represent a practical compromise, exposing more moving body segments to aquatic resistance than shallow immersion while still maintaining sufficient effective loading and attenuating landing stress. In addition, the mechanical resistance during immersed jumping is unlikely to remain constant throughout the movement path, particularly in the mid- and deep-immersion conditions, because buoyancy and drag may vary as body segments move into or out of the water during the eccentric-to-concentric transition. This interpretation is consistent with the present findings and supports immersion depth as an important programming variable because it may influence both the magnitude and phase-specific characteristics of loading during aquatic jumping exercise. It should also be noted that the term plyometric may not imply identical mechanical characteristics across all immersion depths. At chest-level immersion, buoyancy substantially reduces effective body weight and may slow or attenuate the eccentric phase of countermovement-style jumps. Consequently, the deep-immersion condition may have provided a weaker classic stretch-shortening cycle stimulus and a more unloaded, lower-impact, and relatively concentric-oriented jump-training stimulus. This interpretation may partly explain why the deep-immersion group showed smaller mean improvements in several outcomes. Therefore, the present findings should be interpreted as depth-specific responses to aquatic plyometric-style jump training rather than as evidence that each immersion depth provided the same type or magnitude of plyometric stimulus.

Different immersion depths also appeared to be associated with different magnitudes of change in swimming-specific start and turn performance. In the present study, the waist-deep condition showed the most favorable overall changes in 15-m start time and wall contact time, suggesting that this depth may provide a favorable training stimulus for performance adaptations relevant to explosive swimming tasks. Start and turn push-off performance depend on rapid lower-limb force production and effective impulse generation (Puel et al., 2023). Greater immersion may reduce effective loading and weaken the explosive neuromuscular stimulus, whereas shallower immersion may retain loading but lessen the protective unloading effect of water (Waldvogel et al., 2021). Waist-deep immersion may therefore provide a more effective balance between force-oriented stimulus and impact attenuation, which may help explain the favorable changes observed in selected start- and turn-related outcomes. Wall contact time appeared to be more responsive to depth-specific training than 5-m out time, likely because it more directly reflects force generation during the push-off phase (David et al., 2022). By contrast, 5-m out time is an integrated measure that is also influenced by streamline position, underwater trajectory, body alignment, and breakout timing (Tor et al., 2015; Xie et al., 2021). Therefore, although improvements in lower-limb explosive capacity may be relevant to post-turn performance, their contribution cannot be isolated from technical and hydrodynamic factors based on the present outcome measures. Recent exploratory evidence also suggests that jump-based measures may be related to front crawl tumble turn performance, although such associations do not establish a direct mechanical transfer pathway (Febles-Castro et al., 2026). The relationship between improvements in CMJ/SJ performance and start or turn outcomes should therefore be interpreted cautiously. Although CMJ and SJ are commonly used indicators of lower-limb explosive capacity, they are predominantly vertical tasks and do not directly quantify the horizontal or oblique force production required during block take-off or wall push-off in swimming. Thus, the observed improvements in both jump performance and selected start- and turn-related metrics should be interpreted as positive performance adaptations following the intervention, rather than direct biomechanical evidence of transfer from vertical jumping ability to horizontal swimming propulsion. Accordingly, the present findings suggest that immersion depth may influence both lower-limb explosive performance and selected swimming-specific outcomes, but future studies incorporating horizontal kinetic or impulse-based measurements are needed to clarify the biomechanical pathways underlying these adaptations.

From a practical perspective, the present findings do not necessarily indicate that a single immersion depth is optimal for all swimmers or all training phases. Although the waist-deep condition showed favorable responses in this sample, different immersion depths may provide distinct training emphases. Shallow immersion may better preserve effective loading and faster stretch-shortening cycle characteristics, whereas deeper immersion may provide greater unloading and a more concentric-oriented, lower-impact stimulus. Therefore, future studies should examine whether progressive or individualized combinations of immersion depths can produce greater or more targeted adaptations than a single-depth prescription.

Several limitations should be acknowledged. First, this study compared different immersion depths within APT but did not include a non-training control group or a land-based plyometric group. Therefore, the findings are more informative for identifying depth-specific differences within APT than for determining the overall efficacy or superiority of APT relative to other training modes. Second, the sample was small and no formal *a priori* sample-size calculation was performed; therefore, nonsignificant findings, especially for outcomes such as 5-m out time, should be interpreted with caution. Third, only male collegiate swimmers were included, which limits generalizability to female swimmers, youth swimmers, elite swimmers, and other competitive levels. Fourth, although weekly swimming volume and high-intensity swimming sessions were monitored, residual confounding from concomitant swim training cannot be fully excluded. Fifth, the study did not include horizontal jump tests, start-block force or impulse measurements, or underwater wall push-off impulse data. Therefore, the present findings cannot determine whether changes in vertical jump performance were mechanically linked to horizontal or oblique propulsion during swimming starts and turns. Finally, although exploratory pairwise change-score comparisons were adjusted using the Holm-Bonferroni method, multiple secondary outcomes and within-group descriptive comparisons were examined; therefore, these findings should still be interpreted as hypothesis-generating.

## Conclusion

In conclusion, among male collegiate swimmers who continued regular swim training, 8 weeks of additional APT was associated with favorable changes in lower-limb explosive performance and selected start and turn outcomes. The magnitude of adaptation differed by immersion depth. Among the three conditions, the waist-deep condition showed larger mean changes in several outcomes, particularly compared with deep immersion, whereas differences between waist-deep and shallow immersion were less consistent. Because no non-training or land-based comparison group was included, these findings should be interpreted as depth-specific evidence within APT rather than definitive evidence of APT superiority over other training approaches.

## Data Availability

The original contributions presented in the study are included in the article/supplementary material. Further inquiries can be directed to the corresponding author.
